# Establishment of a Novel Mouse Hepatocellular Carcinoma Model for Dynamic Monitoring of Tumor Development by Bioluminescence Imaging

**DOI:** 10.3389/fonc.2022.794101

**Published:** 2022-02-17

**Authors:** Xiangyi Cao, Yulong Zhang, Qianqian Zhou, Sujing Sun, Minwei He, Xiaohui Wang, Ping Ma, Xiaoang Yang, Liping Lv, Linsheng Zhan

**Affiliations:** ^1^ Department of Transfusion Medicine, Institute of Health Service and Transfusion Medicine, Beijing, China; ^2^ Zhengzhou University, BGI College and Henan Institute of Medical and Pharmaceutical Sciences, Academy of Medical Science, Zhengzhou University, Zhengzhou, China

**Keywords:** HCC, mouse hepatocellular carcinoma model, hydrodynamic injection, CRISPR/Cas9, noninvasive monitoring

## Abstract

In this study, a novel mouse model of hepatocellular carcinoma (HCC) was established by simultaneously knocking out Pten and p53 suppressor genes and overexpressing c-Met and △90-β-catenin proto-oncogenes in the livers of mice *via* hydrodynamic injection (HDI). The mutations were introduced using the CRISPR/Cas9 and Sleeping Beauty transposon systems. In this way, a primary liver cancer model was established within six weeks. In addition, macrophages expressing arginase-1(Arg1) promoter coupled with firefly luciferase were engineered for bioluminescence imaging (BLI) of the tumor microenvironment. This novel, rapidly-generated model of primary hepatocellular carcinoma can be monitored noninvasively, which can facilitate not only applications of the model, but also the development of new drugs and treatment strategies of HCC.

## Introduction

Hepatocellular carcinoma (HCC), a highly prevalent malignancy with over 800,000 cases diagnosed annually worldwide, is the second leading cause of cancer-related deaths (1, 2). Early diagnosis and therapy of HCC are important for improvement of the patients’ prognosis. Currently, HCC is diagnosed by imaging (3) and measuring serum alpha-fetoprotein (AFP) levels (4). The traditional imaging techniques used in HCC early diagnosis are computed tomography (CT), positron emission computed tomography (PET) and magnetic resonance imaging (MRI). However, diagnostic has it’s limitation in detecting earlier mini-size cancer (5, 6). Though serum tumor markers, such as AFP and tumor-associated antigens (TAAs), are engaging alternatives for monitoring and early diagnosis of hepatocellular carcinoma, their sensitivities and specificities remain disappointing. As a result, 40% of affected patients are already in the advanced stage of HCC at the time of diagnosis, making surgical treatments impossible. In addition, the first-line treatment for advanced HCC is sorafenib, which provides finite clinical benefit (7). And other first or second-line therapeutic options, such as immune checkpoint inhibitors, have also shown efficacy against HCC recently (8). Thus, the study of early diagnosis and new treatment strategies of HCC remains an attractive issue. Corresponding small animal models play an important role in studies on hepatocellular carcinoma early diagnosis and therapy. Building animal models that are similar to human liver cancer occurrence and development can help us better study the mechanism of liver cancer, while realize early diagnosis and drug development.

Genetically engineered mouse (GEM) models of HCC have been developed based on oncogene knock-in or the knockout of tumor suppressor genes. However, the establishment of such models is an expensive and time-consuming process, and it takes almost three months to establish an HCC mouse model with this technique. Hydrodynamic injection (HDI) is a novel gene transfection technology that can be used to establish a mouse model of liver cancer in a fraction of the time taken by a GEM model. Studies have shown that directly overexpressing c-Met and △90-β-catenin or simultaneously knocking out Pten and p53 in the mouse liver *via* HDI initiates tumorigenesis (9, 10). Since the activation of oncogenes and inactivation of tumor suppressors often synergize during cancer development (11), a murine HCC model was constructed in our study by simultaneously expressing oncogenes and knocking out tumor suppressor genes in the liver using the HDI method.

*In vivo* bioluminescence imaging (BLI) is routinely used to detect gene enhancer/promoter function in tissues or cells through reporter gene constructs (12), and monitor tumor growth, bacterial and viral infection, gene expression and therapeutic responses in real time by fluorescent monitoring devices. Usually, the reporter gene constructs used for BLI is formed by cloning the enhancer/promoter sequence upstream of the luciferase cDNA. Luciferase activities are utilized to reflect the expression of the protein of interest in-time. In addition, during tumor development, immune cells infiltrate in and around tumor tissues through chemotaxis, of which the tumor-associated macrophages (TAMs) are in the majority. TAMs are immune regulatory cells formed by peripheral monocytes under the influence of the tumor microenvironment (13), which can secrete a variety of cytokines and play a key role in promoting cancer cell proliferation, invasion and metastasis (14). The TAMs are classified into the pro-inflammatory M1 macrophages and immunosuppressive M2 macrophages, and M2 polarization is seen during tumor development and characterized by the upregulation of arginase-1 (Arg1) (15). Therefore, combining luciferase expression with activation of the Arg1 promoter is an ideal tool to dynamic monitoring of tumor development.

In this study, a mouse liver cancer model was established quickly by simultaneously overexpressing proto-oncogenes and knocking out tumor suppressor genes through hydrodynamic gene transfection, and sensor macrophages were engineered that could express the luciferase reporter gene under the control of the Arg1 promoter. With the development of HCC, the transgenic macrophages injected in the mouse underwent M1/M2 polarization, followed by Arg1 expression upregulation in macrophages. Thus, the luciferase reporter gene mediated by Arg1 promoter herein was constructed to track M2 polarization activated and thereby the progression of HCC. Using this model, liver cancer development can be monitored based on serological changes and BLI of tumor-infiltrating M2 macrophages.

## Methods

### Experimental Animals

SPF C57BL/6J mice were purchased from Beijing Vital River Laboratory Animal Technology Co., Ltd., weighing 18 ~ 20 g and male. The experimental Animal Center of the Academy of Military Medical Sciences provided the SPF level breeding environment, and animal experiments were approved by the Experimental Animal Ethics Committee of the Academy of Military Medical Sciences 2020-680.

### Plasmids

LentiCRISPRv2 expression plasmid was stored in our lab. PCMV/SB expression plasmid, PT3-EF1a-c-Met expression plasmid, PT3-△90-β-catenin and pGL3-mArg1 promoter/enhancer -31/-3810 expression plasmid were purchased from Addgene Company in USA.

### Construction of Primary Liver Cancer Model in C57BL/6J Mice

The injection volume of normal saline containing plasmids was about 10% of the body weight of each mouse (about 2 mL). The plasmids content was generally about 10 μg/mouse, and the injection time was generally about 3-5 s/time. The amount of plasmids was calculated according to the concentration of extracted plasmids. Plasmids pCMV/SB, PT3-EF1a-c-Met, PT3-△90-β-catenin, LentiCRISPR-sgPten and LentiCRISPR-sgp53 were prepared in the ratio of 1:2:2:2:2, which was dissolved in a proper amount of normal saline, mixed well, and used in an environment free of high temperatures and ultraviolet radiation.

### Western Blot

Western blot was used to detect whether Pten (anti-Pten Cell Signaling, 9559, 1:1000) or p53(anti- p53 Cell Signal,2545, 1:200) was successfully knocked down in recombinant cells and whether c-Met (anti-c-Met Cell Signaling, 8198, 1:1000)or △90-β-catenin (anti-△90-β-catenin Abcam, ab196204, 1:1000)was overexpressed in liver tissue. Lysis buffer RIPA containing protease inhibitor was used for 20 min at 0°C and centrifugated at 12 000 RPM at 4°C for 20 min. Samples were heated at 100°C for 5 min before operation. 25 mg protein was electrophoretized in 10% SDS-PAGE gel and transferred to PVDF membrane. 5% skim milk was incubated overnight with the primary antibody at 4°C After being rinsed with TBS-Tween 20 (0.1%), they were incubated at room temperature for 1 hour with enzymed-labeled goat anti-rabbit or goat anti-rat antibody (1:2000). Finally, chemiluminescence HRP substrate was used to detect the binding of the antibody to the protein.

### Immunohistochemistry (IHC)

Liver tissue was fixed overnight in 4% formalin, embedded in paraffin, sected at 4 μm, and stained with hematoxylin and eosin (H&E) for pathological examination. Oil red staining was used to observe lipids. Immunohistochemical main antibodies included anti-Ki67 (Abcam, ab16667, 1:200), anti-CK19 (Abcam, ab133496, 1:100), anti-p53 (Cell Signaling, 2545, 1:160), Anti-Pten (Cell Signaling, 2545, 1:100), Anti-Met (Cell Signaling, 8198, 1:100) and Anti-△90-β-catenin (anti-△90-β-catenin Abcam, ab196204, 1:1000).

### Detection of TAAs in Liver Tissue

The mRNA expression levels of GNAS, PTCH, NPM1 and PAX5 in recombinant cells were detected by qRT-PCR. Total RNA was extracted using TRIzol reagent (Invitrogen, Carlsbad, CA, USA) according to the instructions. cDNA was then obtained by reverse transcription using Rever Tra AceR qPCR RT Master Mix. PCR amplification was performed using synthetic primers and SYBR-PCR kit (SYBR qPCR Mix).The reaction was carried out in eight consecutive tubes, which were incubated at 95°C for 1 min, 95°C for 15 s, 58°C for 30 s, and 72°C for 30 s. A total of 40 cycles were incubated at 60°C for 5 s and 95°C for 5 s. PCR was performed on a Bio-Rad CFX amplification device. GAPDH mice were used as internal control. Primer sequences of GNAS, PTCH, NPM1 and PAX5 are as follows:

**Table:** 

NPM1	Forward	GTATCTGGACCTGTACCTGATG
NPM1	Reverse	GTGGAGTCGTAGCATATAGTCC
PAX5	Forward	CTGACATCTTCACCACCACGGAAC
PAX5	Reverse	GGTTGTGCTCGCCAAGTCTCG
GNAS	Forward	TGAATCTGAGTCTGATCACGAG
GNAS	Reverse	ATTTCGGTCTCGGATTCGATAT
PTCH1	Forward	TCCATCAGCAATGTCACCGC
PTCH1	Reverse	TCAGTACTGGAGGGAGAACGC

### Serum Collection and ELISA

Serum was collected once a week. The whole blood of mice was collected 100 μL by orbital blood sampling method in 1.5 mL anticoagulant tubes, centrifuged at 2000 RPM for 10 min, and the serum was sucked out and stored at -20℃ for use. For ELISA, the collected serum was balanced to room temperature and diluted twice with the dilution of the sample in the kit, and 20× washing buffer was diluted 1:20 with distilled water. The concentrations of AFP, AST, ALT, GNAS, PTCH, NPM1 and PAX5 in serum were determined respectively. The specific operation steps complied with the instructions for ELISA kit in The Department of Education. Finally, the OD value of each well was measured at the wavelength of 450 nm with a microplate reader.

### The BLI System Monitored the Migration and Distribution of Bone-Marrow Derived Macrophages in Mice

Macrophages were collected and counted, and 5×10^6^ macrophages were mixed in 300 μL PBS, and injected into the hydrodynamic modeling mice for 6 weeks by tail vein, with 5 mice in each group. Migration of bone-marrow derived macrophages was monitored by Xenogen Spectrum imaging at 30 min, 24 h, and 48 h after macrophage infusion. To ensure the imaging quality, the abdomen of mice was depilated to minimize the loss of light signals. Before imaging, mice were intraperitoneally injected with D-fluorescein (150 mg/kg) and anesthetized with isoflurane for 3 min. Imaging was performed to monitor the migration and distribution of macrophages in mice. Vivisection was performed 48 h after macrophage infusion, and macrophages distribution were assayed by BLI in lymph nodes and tissues including inguinal lymph nodes (ILN), axillary lymph nodes (ALN), nodi lymph nodes (NLN), liver lymph nodes (LivLN), mesenteric lymph nodes (MLN), liver tissue, stomach tissue, small bowel tissue, heart tissue, lung tissue, kidney tissue, pancreas tissue. All data were analyzed and processed by *in vivo* imaging software (Xenogen), and standardized calculations were performed for all the data.

### Construction of Arg1EP-Luciferase-GFP/Raw264.7 Engineered Cells

Raw264.7 cells were infected with Arg1EP-Luciferase-GFP lentivirus. Three days after infection, the cells were cultured in DMEM complete medium containing purinomycin 2 μg/mL and 10% FBS. Finally, cells that stably expressed Arg1EP-Luciferase-GFP fusion protein were screened.

### *In Vitro* Detection of the Bioluminescence Activity of Arg1EP-Luciferase-GFP/Raw264.7 Macrophages Induced by Tumor Microenvironment

2×10^5^ Arg1EP-Luciferase -GFP/Raw264.7 cells were inoculated in the upper chamber of a 24-well Transwell culture plate, and the lower layers were added with DMEM medium, DMEM medium containing 25 ng/μL IL-4 and 2×10^5^ Hepa1-6 cells, respectively. After co-incubation of 24 h, 10 μL 15 mg/mL D-Luciferin was added to each well and gently shaken into the Caliper IVIS Lumina II imaging system to detect the bioluminescence activity of Arg1EP-Lucifere-GFP/Raw264.7 cell lines.

### *In Vitro* Detection of Bioluminescence Activity of Arg1EP-Luciferase -GFP/Raw264.7 Co-Incubated With Hepa1-6 Cells

1×10^5^ Arg1EP-Luciferase-GFP/Raw264.7 cells were inoculated in the upper chamber of a 24-well Transwell culture plate, and 2×10^5^ Hepa1-6 cells were inoculated in the lower chamber and co-incubated for 6 h, 12 h, 24 h and 48 h for imaging. (Cells at each time point were set with three multiple wells) 10 uL D-Luciferin was added at the concentration of 15 mg/mL to each well. The bioluminescence activity of Arg1 EP-Luciferase-GFP/Raw264.7 cell line was detected by Caliper IVIS Lumina II imaging system.

### The Migration and Distribution of Arg1EP-Luciferase-GFP/Raw264.7 in Mice Were Monitored by BLI System

Arg1EP-luciferase-GFP/Raw264.7 was collected and counted, and 5×10^6^ Arg1EP-Luciferase-GFP/Raw264.7 was dissolved in 300 μL PBS and mixed. The specific operation was performed using the same method as for monitoring the distribution of macrophages from bone marrow *in vivo*.

### Statistical Analysis

Statistical analysis was performed by GraphPad Prism Software^®^. Two way ANOVA test (Analysis of variance) was used to compare statistical differences among multiple groups. All the experiments were repeated at least three times. Differences among means were considered significant at p <0.05.

## Results

### A Liver Cancer Model Was Successfully Established by Hydrodynamic Gene Transfection

**Figure 1:**
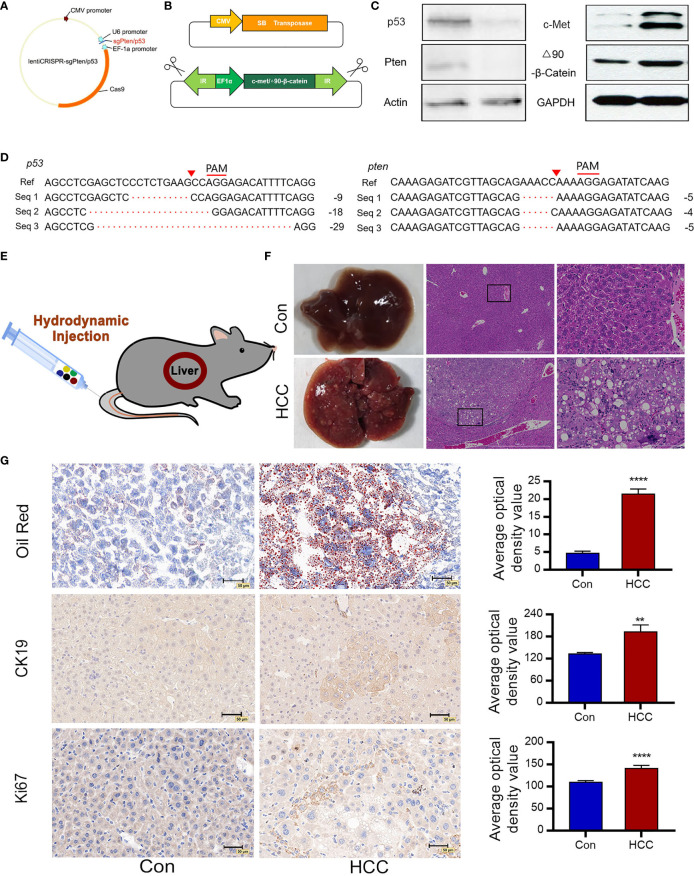
Construction of plasmids and induction of liver tumors following hydrodynamic gene transfection. **(A)** Construction of lentiCRISPRv2/sgRNA plasmids expressing Cas9 and sgPten/p53. **(B)** Schematic diagram of SB transposase-associated plasmids. The SB transposase transfers a gene in the transposon cassette into a host genome. **(C)** Cell genomic DNA sequence of 3T3 cells analyzing deletion mutations of p53 and Pten. Seq 1-3 represent cell genomic DNA read sequences of NIH 3T3 cells knockdown by CRISPR/Cas9. The proto-spacer adjacent motif (PAM) sequences is denoted by red lines. Red triangles indicate the predicted DNA cleavage sites. **(D)** Immunoblots showing loss-of-function of p53 and Pten and overexpressions of c-Met and △90-β-catenin. Actin and GAPDH are used as control. **(E)** Saline containing five plasmids are injected into the tail vein of mice *via* HDI (n=10 for each group). **(F)** Representative macroscopic images of liver tumors observed in C57 mice 6 weeks post-injection and H&E-stained tumor tissue sections indicate large nuclei, multinuclear hepatocytes and reticulated cytoplasm of HCC cells (n=10). **(G)** Oil red O staining demonstrates hepatic steatosis. Ki67 and CK19 immunostaining represent proliferating cells. **P<0.01, ****P<0.0001.

Single-guide (sg) RNA sequences of Pten and p53 were designed and cloned into the CRISPRv2 vector. The schematic diagram of CRISPR/Cas9 related plasmids is shown in **Figure 1A**. The lentiCRISPR-sgPten and lentiCRISPR-sgp53 germ plasmids were then transfected into the NIH 3T3 mouse embryonic fibroblasts, and the genomic DNA of the stably transfected clones was sequenced. After the plasmids were packed into lentiviruses and transduced into the target cells, gene knockout was confirmed at the predicted site before the proto-spacer adjacent motif (PAM) (**Figure 1D**). Compared to the control group, the expressions of Pten and p53 were significantly reduced in the knockout group (**Figure 1C**). In order to achieve the knockout of tumor suppressor genes as well as the long-term stable expressions of oncogenes, the Sleeping Beauty(SB) transposon system was amplified with c-Met and △90-β-catenin genes (**Figure 1B**). The transposon system consisted of a transposon expression vector and a plasmid with an SB transposon box, which contained a promoter, the target gene and a polyA sequence with inverted repeat (IR) sequences. The SB transposable enzyme recognized IRs in the transposable box and catalyzed their translocation from one genomic site to another. The transposon system was transfected into NIH 3T3 cells and the overexpression of c-Met and △90-β-catenin was confirmed by Western blot (**Figure 1C**).

The pCMV/SB, pT3-EF1a-c-Met, pT3-△90-β-catenin, lentiCRISPR-sgPten and lentiCRISPR-sgp53 plasmids were mixed in equal proportions and injected into the tail vein of mice under high pressure. The control groups were injected with pCMV/SB and pT3-EF1α. Hydrodynamic transfection is shown in **Figure 1E**. To further monitor cancer progression, random mice were euthanized every two weeks and their liver tissues were analyzed. In the sixth week, the livers of normal mice appeared healthy with a brown tinge and smooth surface, while those of the experimental group were significantly enlarged, looked dark red due to an excessive amount of blood, and had a filamentous surface with numerous white diffuse tumor masses (**Figure 1F**). Tumor continued to grow and the liver filled the entire abdomen by the eighth week, resulting in abdominal swelling. Hematoxylin and eosin (H&E) staining of the malignant liver tissues showed a three-fold to four-fold increase in the size of hepatocytes and obvious crowding of hepatocyte cords. In addition, the hepatocytes had multiple cytoplasmic vacuoles and vacuolar degeneration along with inclusion bodies in the nucleus. Some cells had enlarged or multiple nuclei, and a number of enlarged nuclei were irregular, which was a sign of obvious regeneration of liver cells. In contrast, the hepatocytes in the normal liver were aligned **(** **Figure 1F** **)**. Oil red staining showed a significant increase in the number of lipid droplets in the hepatocytes and hepatic steatosis (**Figure 1G**). CK19 was commonly expressed in bile duct epithelial cells, and absent from the mature liver cells. Tumor proliferating antigen Ki67 was highly expressed in numerous human solid tumors, which was a marker of cell proliferation and was correlated with patient prognosis. Numerous CK19 and Ki67 positive oval cells were observed in the liver tumors, indicating active proliferation of tumor cells and a high degree of malignancy (**Figure 1G**).

### Serological Detection of Primary Mouse Hepatocellular Carcinoma

**Figure 2:**
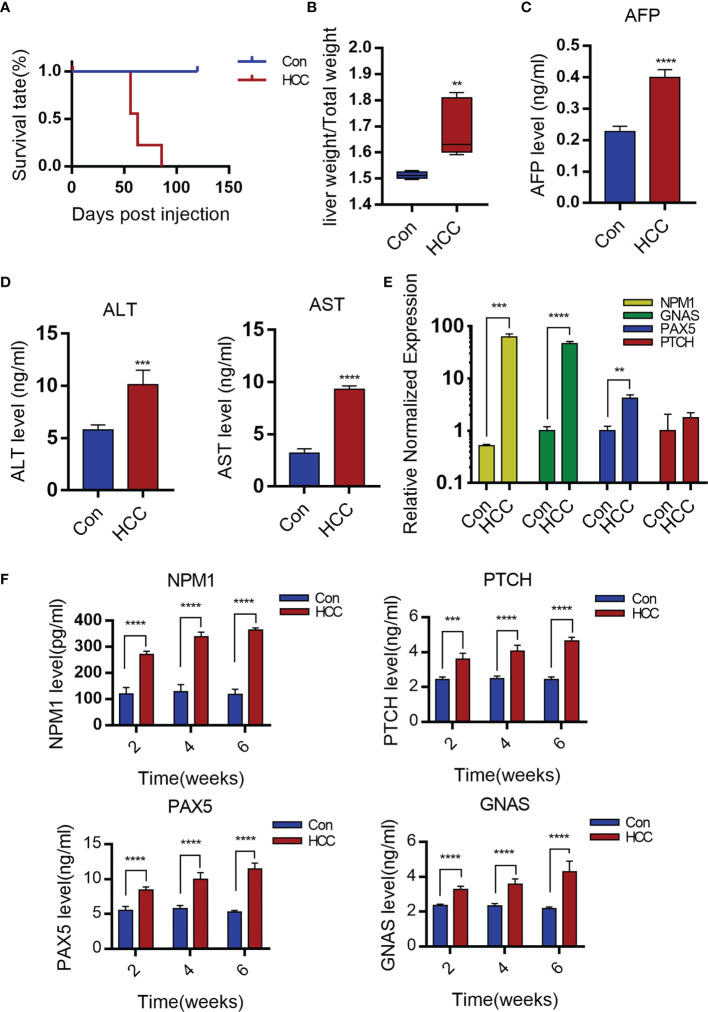
Serological detection of primary mouse hepatocellular carcinoma. **(A)** The survival rate of mice after establishment of liver cancer model. **(B)** Liver weight relative to the whole-body weight of mice 6-week post injection. Data represent mean ± S.D. n=5. **(C, D)** AFP, ALT and AST levels of serum 6 weeks post- injection. Mean ± S.D; n=3, Statistical significance determined with t test; **P<0.05, ***P<0.001, ****P<0.0001; compared with the control. **(E)** Expression levels of GNAS, NPM1, PAX5 and PTCH genes in mouse liver tissue. Mean ± S.D; n=3, Statistical significance is determined with t test; ** P<0.01, ***P<0.001, ****P<0.0001; Compared with the control. **(F)** Serum levels of NPM1, GNAS, PAX5 and PTCH are time-dependence increases. Mean ± S.D; n=3, Statistical significance is determined with t test; ***P<0.001, ****P<0.0001.

There was no significant change in the survival rates in the first eight weeks after injection, but 50% HCC mice died after eight weeks, all mice with the HCC died at the third month (**Figure 2A**). The ratio of liver weight to body weight was also markedly higher in the tumorigenic group than in the control group (**Figure 2B**). The liver cancer model was verified six weeks later by analyzing serum AFP levels. AFP is synthesized in large amounts in fetal hepatocytes and undifferentiated liver cancer cells, and is therefore a specific marker of liver cancer. The AFP levels were significantly higher in the experimental versus the control group, indicating the success of liver cancer modeling (**Figure 2C**). In addition, the serum levels of alanine aminotransferase (ALT) and glutamic-oxaloacetic aminotransferase (AST) were increased significantly in the liver cancer group, suggesting considerable liver damage (**Figure 2D**).

The production of TAAs is a humoral immune response in HCC patients, autoantibodies to TAAs may be detected even in patients’ serum at early-stage (16–18). Wang et al. identified antibodies specific to 11 TAAs, including TP53, MSH2, mouse G-protein α subunit (GNAS), Pten, mouse pile-protein 5 (PAX5), GNA11, mouse patching homologue (PTCH), IDH1, SRSF2, mouse nucleolar phosphate 1 (NPM1) and Survivin by screening the sera of a large number of liver cancer patients. In our study, it was found that the PTCH, PAX5, GNAS and NPM1 mRNAs were significantly upregulated in liver tumors compared to healthy liver tissues (**Figure 2E**). Moreover, the sera of tumor-bearing mice showed increased titers of antibodies specific to NPM1, GNAS, PAX5 and PTCH at weeks 2, 4 and 6. In contrast, no significant changes were observed in the control group (**Figure 2F**).

### Detection of Proto-Oncogenes Overexpression and Tumor Suppressor Genes Knock Down in Liver Tumors

**Figure 3:**
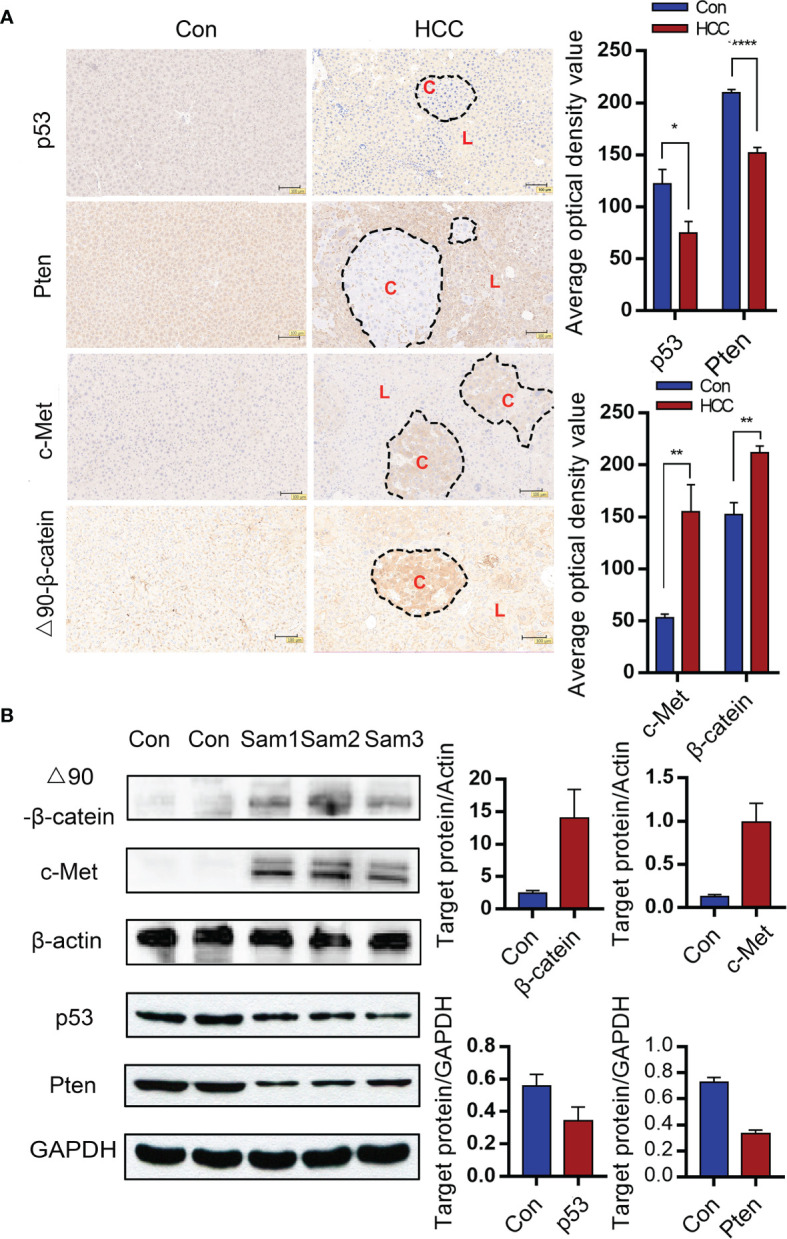
Gene alterations in the liver tumor nodules. **(A)** Immunohistochemical (IHC) of liver tumor tissues indicating knockout of Pten and p53 and overexpressions of c-Met and △90-β-catenin. (C-carcinoma, L-liver) (Magnification: 100x) Mean ± S.D; n=3, Statistical significance determined with t test; *P<0.05, **P<0.01, ****P<0.0001. **(B)** Immunoblot showing overexpressions of c-Met and △90-β-catenin protein and decreased levels of p53 and Pten proteins in the mouse liver. *P<0.05, ** P<0.01, ****P<0.0001.

To find out whether liver tumorigenesis resulted from oncogene overexpression and loss of tumor suppressor genes, the expressions of corresponding proteins in liver tissues were analyzed. Immunohistochemistry (IHC) was used to observe the Sleeping Beauty translocation enzyme incorporated into mice liver cells that expressed corresponding proteins in the genome. Negative signals suggested suppressor genes knocked-down in the circled carcinoma area of the two upper panels. Besides, positive signals were observed in the cytoplasma of hepatoma cells, which showed c-Met and △90-β-catenin overexpressions in the circled carcinoma area of the two bottom panels (C-carcinoma, L-liver). In contrast, the control mice did not show any antibody positive staining (**Figure 3A**). On the basis of serial sections analysis, it was showed that some tumors stained positively for c-Met and β-catenin as well as negatively for Pten and p53 in similar locations, which suggested that part of the transformed hepatocytes had 4 intended changes (**Supplementary Figure 1**). The protein expressions of p53, Pten, c-Met and △90-β-catenin were verified by Western blot. Grayscale statistics were performed on the results as shown in **Figure 3B**. The deletion of endogenous p53 and Pten by the CRISPR system was confirmed by sequencing the DNA extracted from liver nodules 6 weeks after modeling. However, p53 and Pten genes were intact in the livers of control mice (**Supplementary Figure 2**).

### Arg1 Was Highly Expressed in the Primary Liver Tumor Nodules

**Figure 4:**
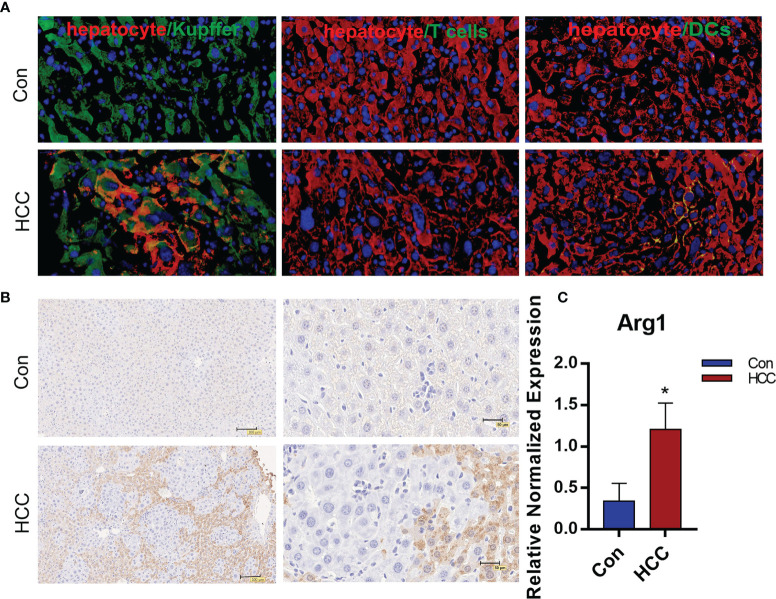
The distribution of immune cells and expression of Arg1 in liver tumors. **(A)**. Distribution of macrophages, T cells and DCs in tumor tissues. The macrophages labeled with Alexa Fluor594, hepatocytes with FITC and the nuclei counterstained with DAPI in the first panel. T cells and DCs with FITC, hepatocytes with Alexa Fluor594, the nuclei counterstained with DAPI in the second and third panels. **(B)** Arg1 expression in the mouse liver. **(C)** Arg1 mRNA levels in Kupffer cells. Mean ± S.D; n=3, Statistical significance is determined with t test; *P<0.05.

**Figure 5:**
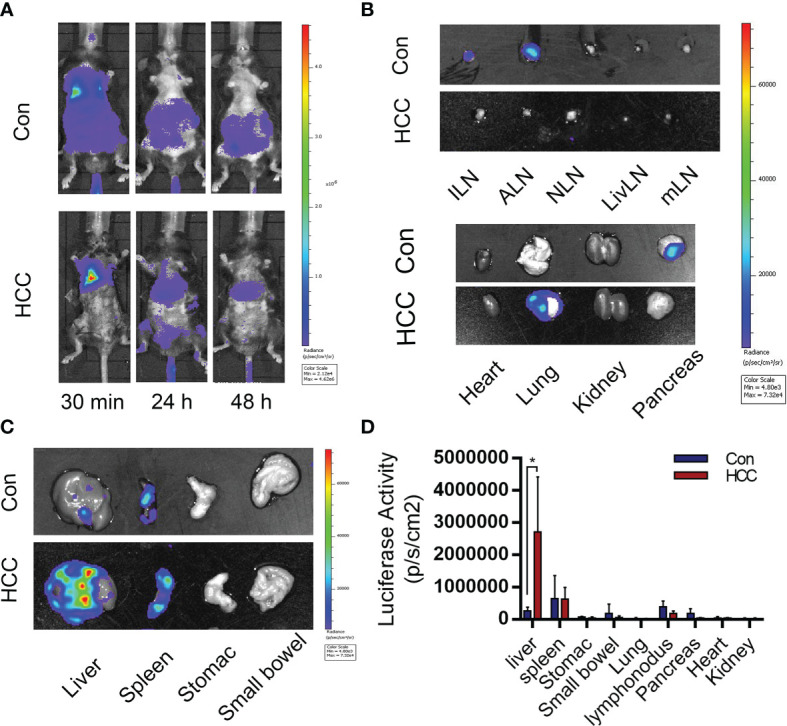
Distribution of bone marrow-derived macrophages (BMDMs) in the control and tumor-bearing mouse model. **(A)** BMDMs’ migration from 30 min to 48 h after cell injection. **(B, C)** BMDMs are mainly distributed in the tumor-bearing mice liver and spleen. **(D)** Statistical analysis of the luciferase activity in major organs. Mean ± S.D; n=3, Statistical significance is determined with t test; *P<0.05.

The infiltration of immune cells in liver tumors was analyzed by immunostaining the tissue sections with specific markers. The Kupffer cells were the most abundant, followed by T cells and dendritic cells (**Figure 4A** and **Supplementary Figure 3**). Arg1 was highly expressed around tumor tissues, but was hardly observed in healthy liver tissues (**Figure 4B**). The Kupffer cells were isolated by liver perfusion, which confirmed the significantly higher expression of Arg1 in tumor-bearing mice compared to the controls (**Figure 4C**). These results indicated that the Kupffer cells played an important role in the development of primary liver cancer. To add to our evidence, bone marrow-derived macrophages (BMDM) were adoptively transferred from Luc+ reporter mice into the control and tumor-bearing mice by tail vein infusion, and their distribution and migration were monitored by *in vivo* BLI. As shown in **Figure 5A**, intense bioluminescence was observed in the lungs within 30 min of infusion in both the control and tumor-bearing mice. After 24h, bioluminescence in the experimental mice was concentrated in the liver, whereas only small intestines showed weak bioluminescence in the control mice. The Luc+ macrophages remained in the liver of tumor-bearing mice after 48h, while those in the control group migrated to the spleen and small intestines (**Figure 5A**). *Ex vivo* BLI of the inguinal lymph nodes (ILN), axillary lymph nodes (ALN), submaxillary lymph nodes (NLN), hepatic lymph nodes (LivLN), mesenteric lymph nodes (MLN), and other organs or tissues after 48h also confirmed that the macrophages were mainly distributed in the livers of tumor-bearing mice, whereas those in the control group mainly homed to the secondary lymph nodes and the spleen (**Figures 5B, C**). Bioluminescence statistics of major organs are shown in **Figure 5D**, indicating that the bioluminescence intensity of the liver was the strongest. These findings suggested that the infiltration of macrophages into the liver tumor microenvironment led to their polarization to the Arg1-expressing M2 phenotype.

### Macrophage Sensors Were Constructed to Monitor Liver Tumor Development *In Vitro*

**Figure 6:**
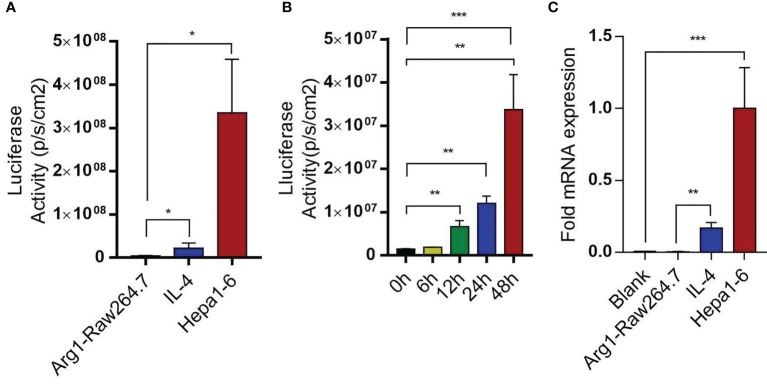
Arg1EP-Luciferase-GFP/Raw264.7 macrophage sensors in tumor microenvironment. **(A)** Luciferase activity of macrophage sensors in different tumor microenvironments. **(B)** In the condition of co-incubation of Hepa1-6 showing increased luciferase activity of macrophage sensors within 48 h. **(C)** Changes in Arg1 mRNA levels after IL-4 inducein and in tumor microenvironment. Mean ± S.D; n=3, Statistical significance is determined with t test; *P<0.05, ***P<0.001.

To test the above hypothesis, Raw264.7 macrophages were transducted with Arg1EP-Luciferase-GFP lentivirus and incubated with Hepa1-6 cells. The bioluminescence intensity of Arg1EP-Luciferase-GFP/Raw264.7 cells also increased significantly when cultured in the presence of IL-4 compared to the untreated controls, while bioluminescence intensity of Arg1 macrophages co-incubated with Hepa1-6 was the strongest in the tumor microenvironment. (**Figure 6A** and **Supplementary Figure 4**). The bioluminescence intensity of the macrophages increased within 24h of co-culture and peaked at 48h (**Figure 6B**). Likewise, Arg1 mRNA levels increased significantly in the Arg1EP-Luciferase-GFP/Raw264.7 cells after 24h of co-culture with Hepa1-6 cells, as well as following IL-4 induction (0.1678 ± 0.02247), compared to the control group (P <0.05; **Figure 6C**).

### Macrophage Sensors Were Used to Monitor Liver Tumor Development

**Figure 7:**
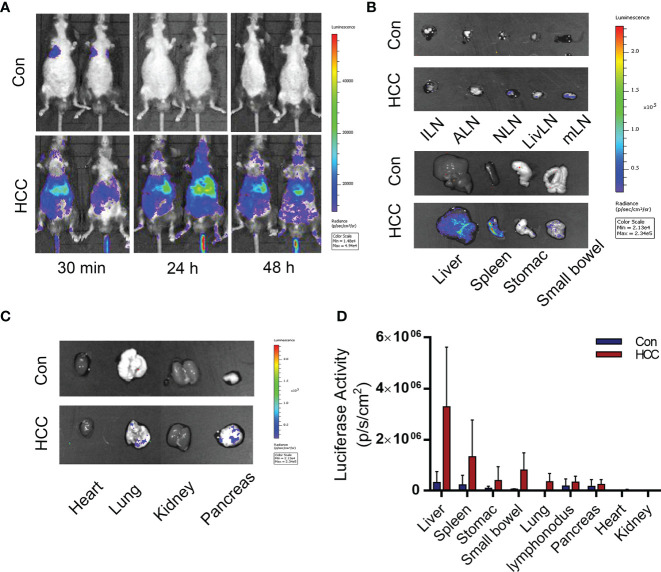
Distribution of Arg1EP-Luciferase-GFP/Raw264.7 in the control and tumor-bearing mouse model. **(A)** Distribution of Arg1EP-Luciferase-GFP/Raw264.7 in mice from 30 min to 48 h point after cell injection. **(B, C)** Arg1EP-Luciferase-GFP/Raw264.7 mainly distributed in the liver and spleen of tumor-bearing mice. **(D)** Statistical analysis of the luciferase activity in major organs.

On a prior basis, the macrophage sensor was used to monitor the hepatocellular carcinoma model. Arg1EP-Luciferase-GFP/Raw264.7 cells were injected into the tumor-bearing mice for 6 weeks through the tail vein. The macrophages were tracked by *in vivo* BLI and the intensity was used to assess the progression of tumors (**Figure 7A**). As shown in **Figure 7B**, the macrophages that had been injected into the tumor-bearing mice initially migrated to the lungs and livers within 30 min before settled in the livers by 24 h, and remained in the livers even after 48 h. In contrast, the macrophages in the control group were accumulated in the lungs within 30 min, but were not detected after 24 h. The mice were euthanized 48 h after injection, and their tissues (see previous section) were imaged. As shown in **Figures 7B, C**, the bioluminescence signals were concentrated in the livers and spleens of the tumor-bearing mice, but no signals were detected in any of the tissues of control mice. The quantitative results of light signal in major organs showed that HCC significantly enhanced the M2 polarization of macrophages, with the strongest signal detected in the liver, indicating the macrophage sensor can indeed reflect the concurrence of liver cancer. In addition, a moderate signal intensity could also be detected in the spleen of HCC mice, though dramatically lower than that in the liver. We speculate that those extrahepatic macrophages may be polarized in the liver and then migrated to other organs, such as the spleen. Secondly, HCC may also elicit systemic changes of internal environment, leading to M2 polarization of macrophage sensors outside the liver and thus being monitored. Anyway, the highest signal in the liver suggests that the macrophage sensors are moderately specific to follow HCC progression (**Figure 7D**).To summarize, sensors of M2 polarization of macrophage sensors can be used to track tumor growth, as well as the formation of the tumor microenvironment.

## Discussion

A primary liver cancer model was established in mice by simultaneously knocking out Pten and p53, and overexpressing c-Met and △90-β-catenin by hydrodynamic gene transfection. This model was able to simulate the initiation and development of human primary liver cancer, but was technically unchallenging. In addition, this novel liver cancer model could be used as a promising tool for screening diagnostic markers and therapeutic targets. Tward et al. established a liver cancer model by overexpressing both c-Met and △90-β-catenin in the Sleeping Beauty transposase system, and detected tumor formation after 3 months (19). Likewise, Xue et al. induced HCC in FVB mice after 3 months by knocking out Pten and p53 using the CRISPR/Cas9 system (20). To the best of our knowledge, this was the first time that a primary mouse liver cancer model had been established by simultaneously overexpressing c-Met and △90-β-catenin, and knocking out Pten and p53, which could observe extensive tumor growth within 6 weeks. C-Met is a tyrosine kinase that is widely expressed in HCC cell lines and activates △90-β-catenin, which is frequently mutated in various cancers. Mutations and aberrant overexpression of △90-β-catenin is closely related to tumor development and prognosis (21). In addition, c-Met and the mutated Δ90-β-catenin synergistically promote HCC (22). The tumor suppressor Pten inhibits the PI3K/AKT pathway, which regulates cell proliferation, apoptosis, protein synthesis and glucose metabolism, and promotes tumor progression (23). Pten is mutated or deleted in multiple cancers, and loss of Pten in hepatocytes leads to lipid accumulation (24). The tumor suppressor p53 plays a key role in inducing cellular senescence and death (25). Deletion of p53 is the most common genetic aberration in liver cancer, and promotes HCC tumorigenicity and lung metastasis *via* mTOR/Pten/Akt pathway activation. However, neither sgPten nor sgp53 can induce tumor formation alone, whereas simultaneous mutation of both can significantly accelerate liver cancer progression in mice (10).

Liver carcinogenesis was also confirmed in terms of pathological and serological indices. The hydrodynamic gene transfection technology simulates the spontaneity of primary liver cancer, which accelerates tumor induction. Previous studies have shown that early-stage liver cancer is characterized by the appearance of anti-TAA antibodies in the serum, which are potentially useful biomarkers for early diagnosis. Consistent with this finding, we detected a time-dependent increase in the serum levels of NPM1, GNAS, PAX5 and PTCH in tumor-bearing mice. Therefore, it is possible to determine the course of cancer development according to the changes of these serum biomarkers.

Early imaging diagnosis has limited effectiveness. For instance, CT is relatively expensive and is of limited diagnostic value, whereas PET can detect nothing more than tumors larger than 200 mm^3^ and measuring more than 7 mm in diameter. In addition, MRI is time consuming, has low spatial resolution, and is therefore not effective for early tumor detection. BLI is a versatile and sensitive technique for longitudinal monitoring of cancer progression using cells that express luciferase reporter genes. Gambhir et al. used an arginase promoter to drive reporter gene expressions in colorectal and breast mouse tumor models, and the engineered macrophages after polarization could be detected by BLI and luciferase measured in the blood (26). Our study built a new approach to monitor hepatocellular carcinoma models using an Arg1 luciferase reporter plasmid. It is valuable to further studies regarding new drugs and treatment strategies of HCC, especially in evaluating the efficacy of immunotherapy in treating HCC.

The primary mouse liver cancer model established *via* hydrodynamic gene transfection technology is simple to operate, inexpensive and can better fit the occurrence and development of human liver cancer. It can not only provide a reliable animal model for the screening of markers for the early detection of HCC, but also facilitate the research on the mechanism of HCC and the development of anti-tumor drugs. In the early stage of tumor formation, it is impossible to intuitively observe the tumorigenesis of a mouse liver. By combining this imaging approach with engineered immune cell, the formation of the tumor microenvironment of a mouse liver can be determined in the early stage of tumor formation. As a result, the damage caused by vivisection can be prevented and the mouse liver cancer model used more efficiently, which will contribute to drug research and development while facilitating the treatment of primary liver cancer.

## Conclusion

In our study, a primary mouse liver cancer model was established within six weeks by overexpressing the proto-oncogenes c-Met and △90-β-catenin, and knocking out the tumor suppressors Pten and p53 by HDI. Antibodies specific to TAAs such as PTCH, PAX5, GNAS and NPM1 increased in the mouse liver and serum in a time-dependent manner, and therefore can be used as early immunodiagnostic markers of HCC. A reporter plasmid was also constructed to track the Arg1-overexpressing M2 macrophages during liver carcinogenesis through BLI.

## Data Availability Statement

The original contributions presented in the study are included in the article/**Supplementary Material**. Further inquiries can be directed to the corresponding authors.

## Ethics Statement

The animal study was reviewed and approved by Experimental Animal Ethics Committee of the Academy of Military Medical Sciences 2020-680.

## Author Contributions

LL and LZ contributed to conception and design of the study. YZ, QZ, SS and MH organized the database. XY, XW, and PM performed the statistical analysis. XC wrote the first draft of the manuscript. All authors contributed to the article and approved the submitted version.

## Funding

This work was supported by the Grant 2018ZX10302205 from the National Science and Technology Major Projects of China.

## Conflict of Interest

The authors declare that the research was conducted in the absence of any commercial or financial relationships that could be construed as a potential conflict of interest.

## Publisher’s Note

All claims expressed in this article are solely those of the authors and do not necessarily represent those of their affiliated organizations, or those of the publisher, the editors and the reviewers. Any product that may be evaluated in this article, or claim that may be made by its manufacturer, is not guaranteed or endorsed by the publisher.
