# Supplementary material for: Establishment of a Novel Mouse Hepatocellular Carcinoma Model for Dynamic Monitoring of Tumor Development by Bioluminescence Imaging
Source: Front Oncol. 2022 Feb 17;12:794101. doi: 10.3389/fonc.2022.794101 (PMC8891637; doi:10.3389/fonc.2022.794101)
Supplement: Supplementary file 1 [file DataSheet_1.docx]

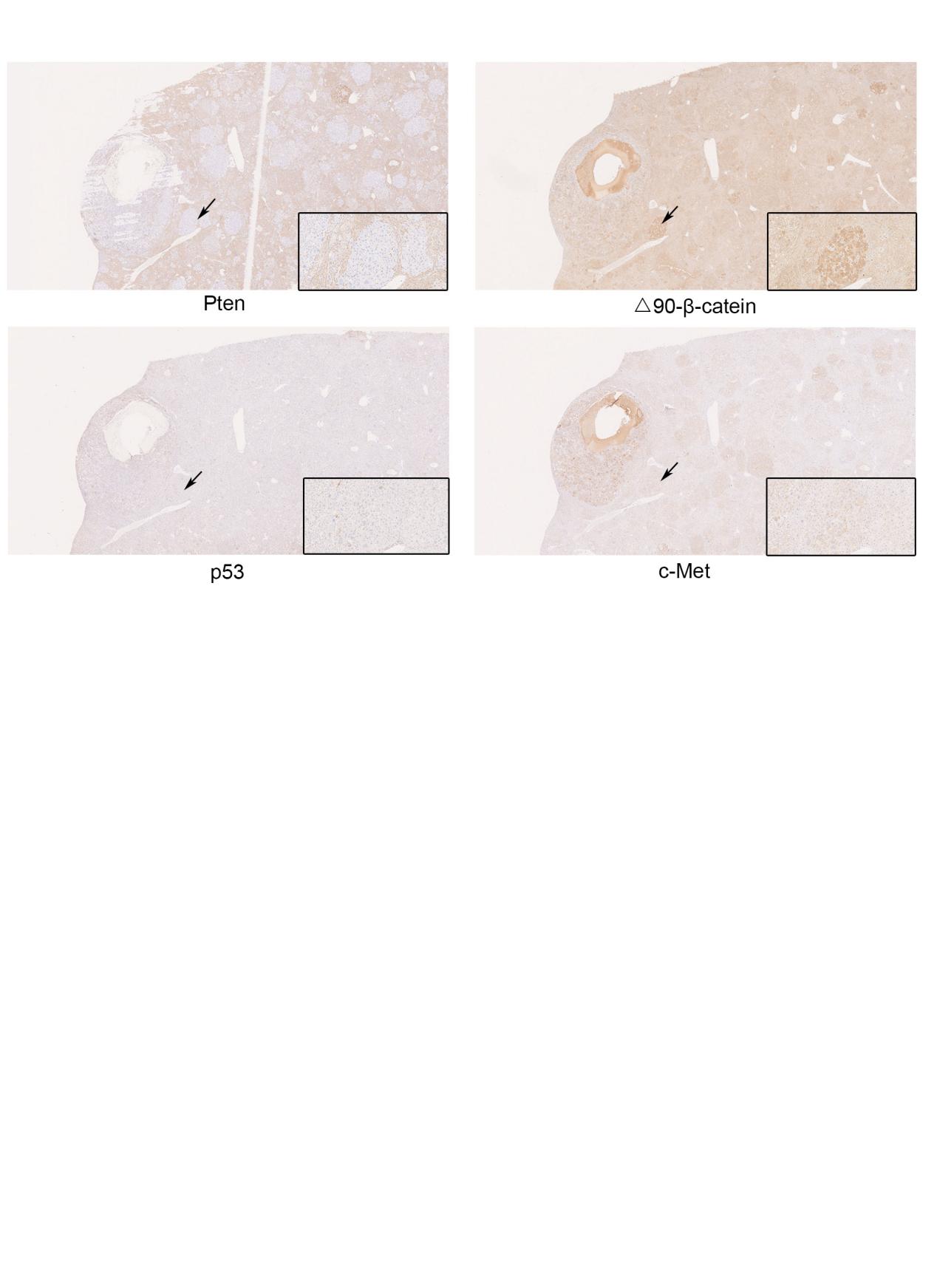


Supplementary Figure 1. Histology showing positive staining for c-Met and β-catenin as well as negative for Pten and p53 of some tumors (Black arrows)


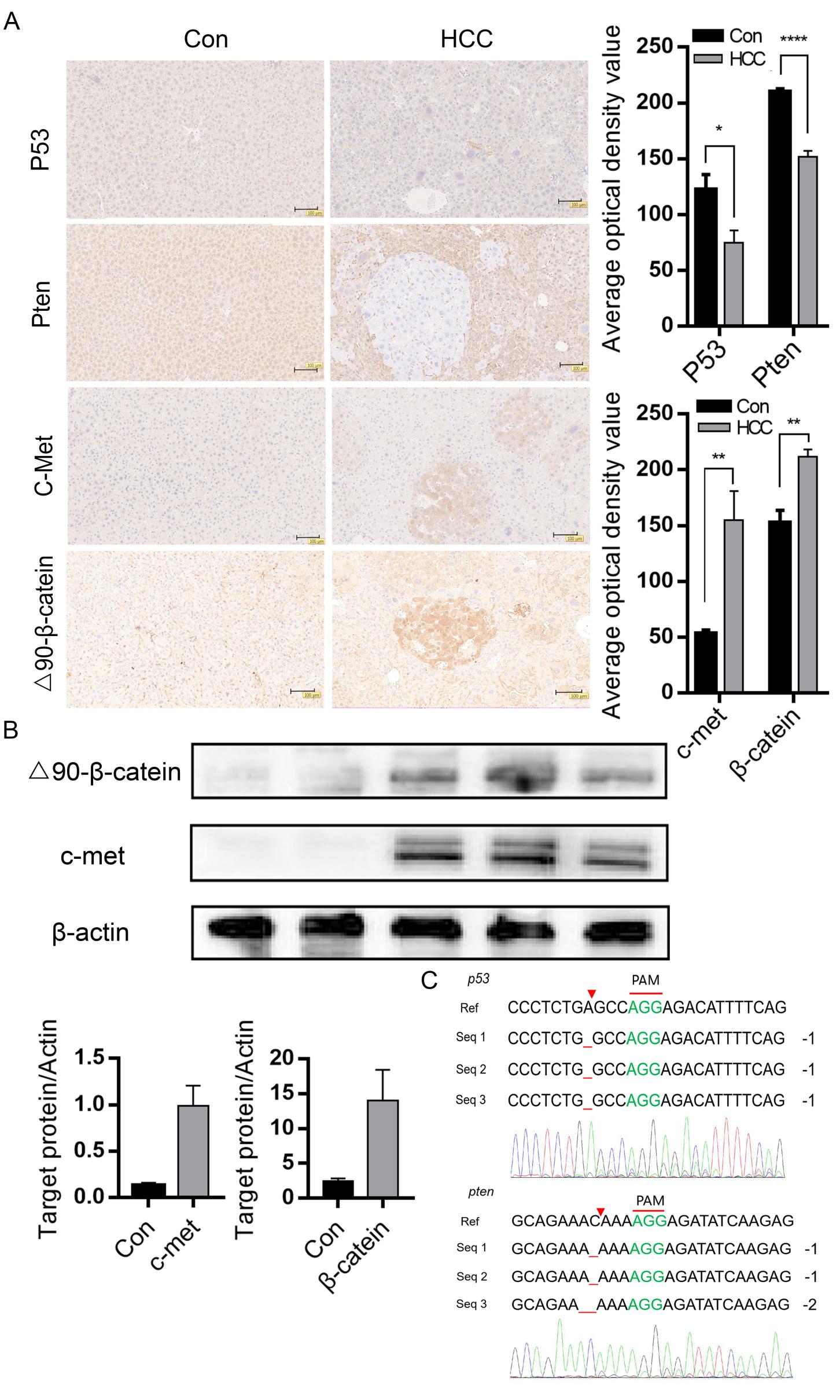


Supplementary Figure 2. The sequence of the liver DNA showing the knockout of Pten and p53 genes in mouse liver. Seq 1-3 represent genomic DNA knockout of mice liver. The proto-spacer adjacent motif (PAM) sequences are denoted by red lines. Red triangles indicate the predicted DNA cleavage sites.


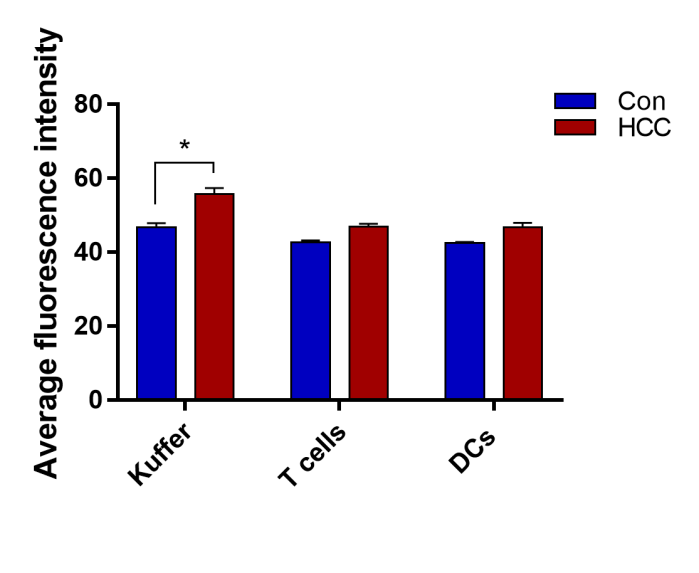


Supplementary Figure 3. Distribution of macrophages, T cells and DCs in tumor tissues. The infiltration of immune cells in liver tumors is analyzed by immunostaining the tissue sections with specific markers. The Kupffer cells are the most abundant, followed by T cells and DCs. Mean ± S.D; n=3, Statistical significance is determined with t test; * P<0.05.


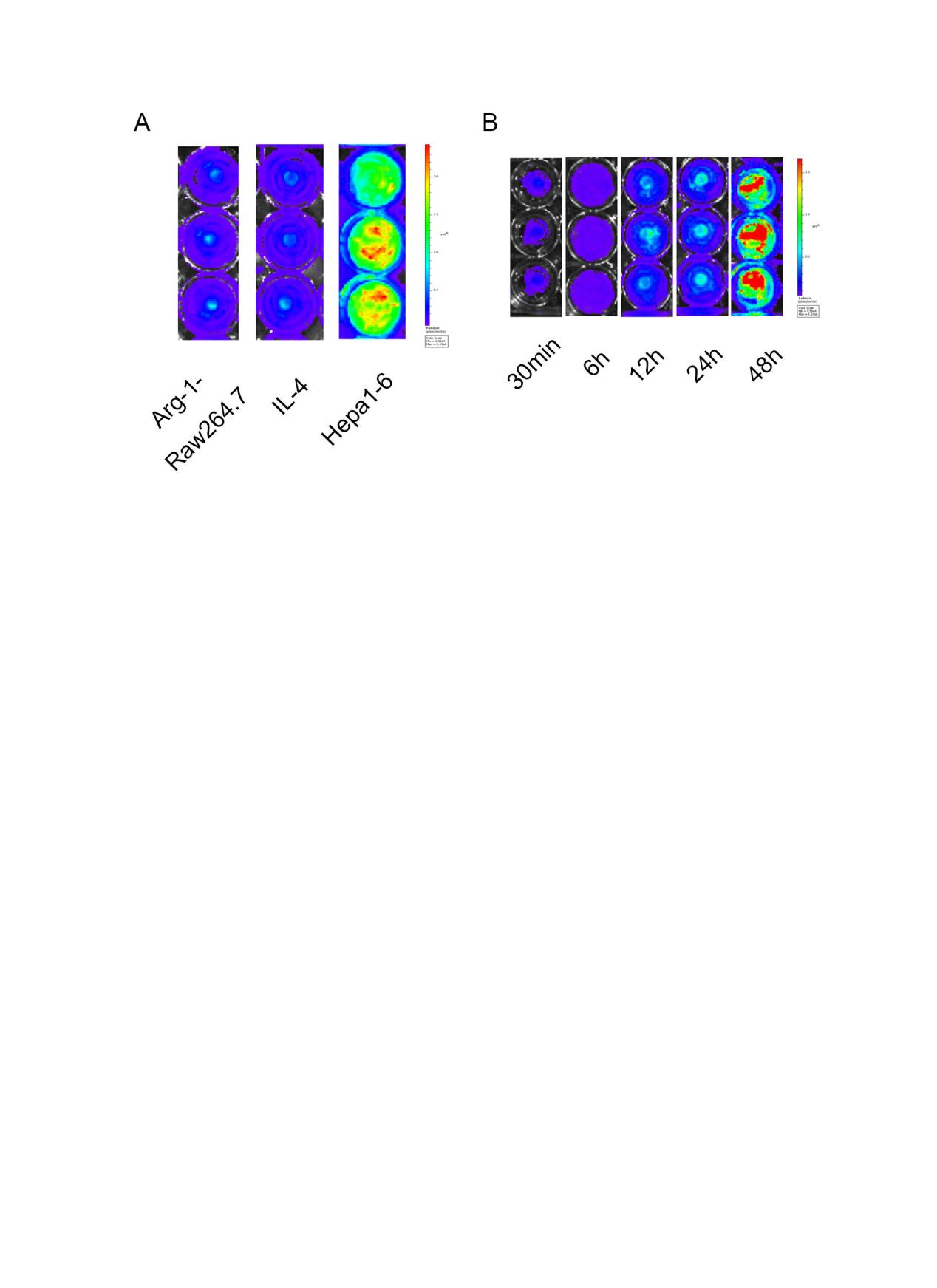


Supplementary Figure 4. Arg1EP-Luciferase-GFP/Raw264.7 macrophage sensors in the tumor microenvironment. (A) BLI of Arg1 macrophages co-incubated with Hepa1-6 is the strongest in the tumor microenvironment. (B) BLI showing increased luciferase in the tumor microenvironment within 48 h.
